# Supplementary material for: Inverse-designed silicon carbide quantum and nonlinear photonics
Source: Light Sci Appl. 2023 Aug 22;12:201. doi: 10.1038/s41377-023-01253-9 (PMC10444789; doi:10.1038/s41377-023-01253-9)
Supplement: Supplementary file 1 — Supplementary Information [file 41377_2023_1253_MOESM1_ESM.pdf]

Supplementary Information for Inverse-designed Silicon Carbide Quantum and Nonlinear Photonics

Joshua Yang[1], Melissa A. Guidry[1], Daniil M. Lukin[1], Kiyoul Yang[1,2], and Jelena Vučković[1,*]
[1]E.L.Ginzton Laboratory, Stanford University, Stanford, CA, USA.
[2]John A. Paulson School of Engineering and Applied Sciences, Harvard University, Cambridge, MA, USA.
*Corresponding author: jela@stanford.edu

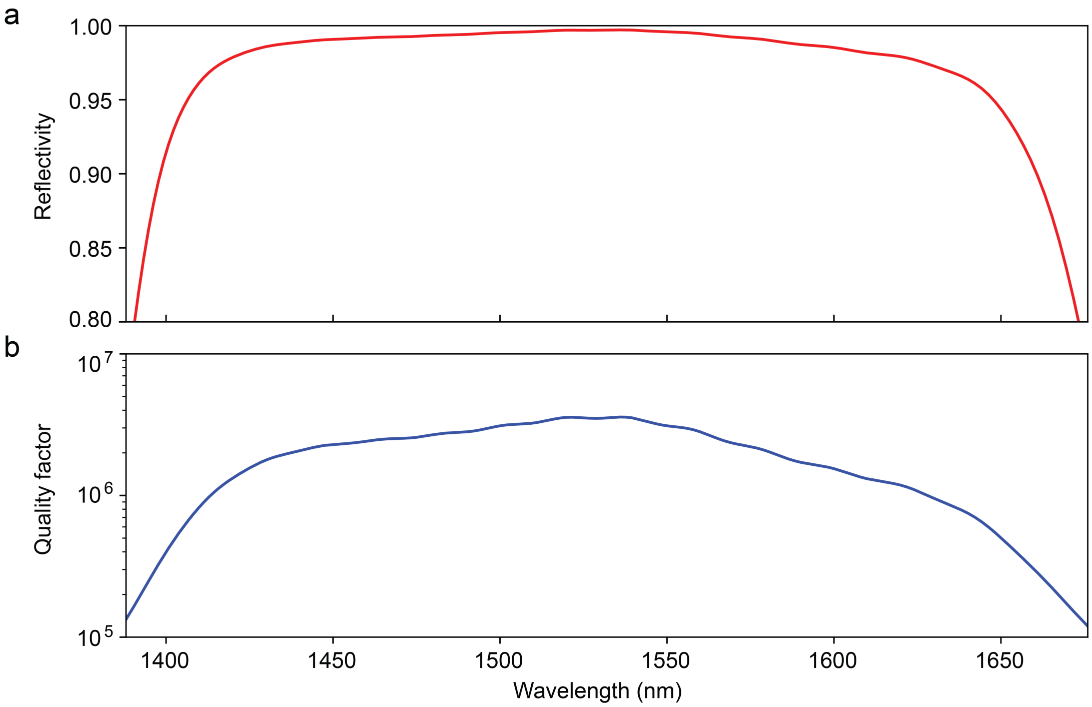

Fig. S1. **Inverse-designed silicon carbide nanoreflector** (**a**) Simulated reflectivity of the inverse-designed reflector, with peak reflectivity of 99.7%. (**b**) Quality factors of a 3 mm FP cavity, corresponding to simulated reflectivities, with a waveguide propagation loss of 0.08 dB/cm.
